# Case report: A rare case of neurocytoma of the Vth cranial nerve

**DOI:** 10.3389/fonc.2024.1438011

**Published:** 2024-09-27

**Authors:** Yongping Gui, Fanghua Zhou, Bin Li, Bin Wu, Xingen Huang, Zhaomu Zeng, Shuhong Mei

**Affiliations:** ^1^ Department of Neurosurgery, Jiangxi Provincial People’s Hospital, The First Affiliated Hospital of Nanchang Medical College, Nanchang, China; ^2^ Department of Anesthesiology, Ji’an Central People’s Hospital, Ji’an, China; ^3^ Department of Neurosurgery, Ji’an Central People’s Hospital, Ji’an, China

**Keywords:** extraventricular neurocytoma, trigeminal nerve, immunohistochemistry, origin and pathological basis, case report

## Abstract

We report a case of neurocytoma originating from cranial nerve V. A 53-year-old female patient presented with a 20-day history of right frontotemporal facial paresthesia and pain. Magnetic resonance imaging (MRI) showed a 2.5-cm × 1.4-cm “dumbbell” enhancing lesion located in the cisternal segment of cranial nerve V with extension into Meckel’s cave, and the signal characteristics were suggestive of trigeminal neurinoma. The lesion was resected through a subtemporal middle cranial fossa approach. Intraoperative findings revealed that the tumor originated from the cisternal segment of cranial nerve V and extended into Meckel’s cave through the trigeminal foramen. No dural attachment was found. The tumor was debulked using sharp dissection and bipolar cautery under the microscope. Extraventricular neurocytomas (EVNs) are extremely rare tumors of the central nervous system. To date, only two cases of neurocytomas arising from cranial nerve VIII have been described. This paper summarizes the clinicopathological features of a case of neurocytoma originating from the cisternal segment of cranial nerve V with extension into Meckel’s cave and expounds the relevant diagnoses and treatments, which may provide a practical clinical basis and experience for the diagnosis and treatment of EVN in the future.

## 1 Introduction

Neurocytoma is a rare neoplasm of the central nervous system (CNS) and shows a propensity to occur within the lateral ventricles, known as central neurocytoma (CN). In contrast, neurocytoma arising outside the ventricles, known as extraventricular neurocytoma (EVN), is an extremely rare neuronal neoplasm that has not been well-characterized (1). In 1989, Ferrol et al. (2) reported a case of extraventricular tumor. Gangaspero et al. (3) frist proposed the concept of EVN in 1997. The incidence rate of EVN is about 0.13%, and only more than 100 cases have been described to date. As reported, EVNs occur in heterogeneous locations, most commonly in the cerebral hemisphere (particularly the frontal and temporal lobes), but also in the thalamus, sellar region, cerebellar hemispheres, brainstem, and rarely in an extracranial locations of the spinal cord and cranial nerves (4). So far, only two cases of EVNs arising from the VIII cranial nerve have been described (5, 6). The present study is the first to report a case of trigeminal neurocytoma due to the rare involvement of cranial nerves by EVNs.

## 2 Case report

**Figure 1:**
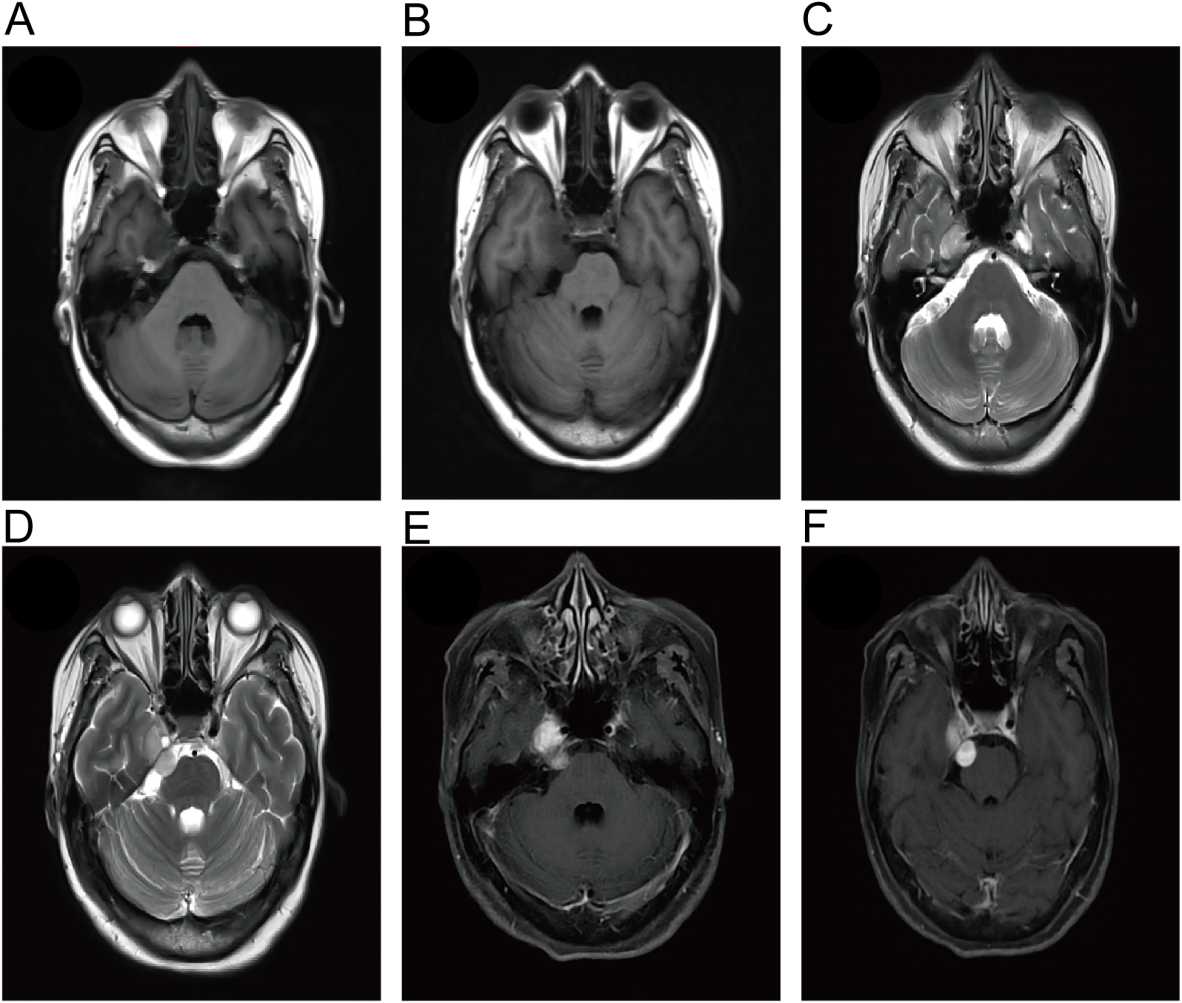
Magnetic resonance imaging (MRI) of the patient showed an enhancing lesion in the cisternal segment of the trigeminal nerve that protruded into Meckel’s cavity. The lesions showed hypointense on T1-WI **(A, B)** and hyperintense on T2-WI **(C, D)**. **(E, F)** Gd-enhanced MRI showed a well-circumscribed dumbbell-like mass.

**Figure 2:**
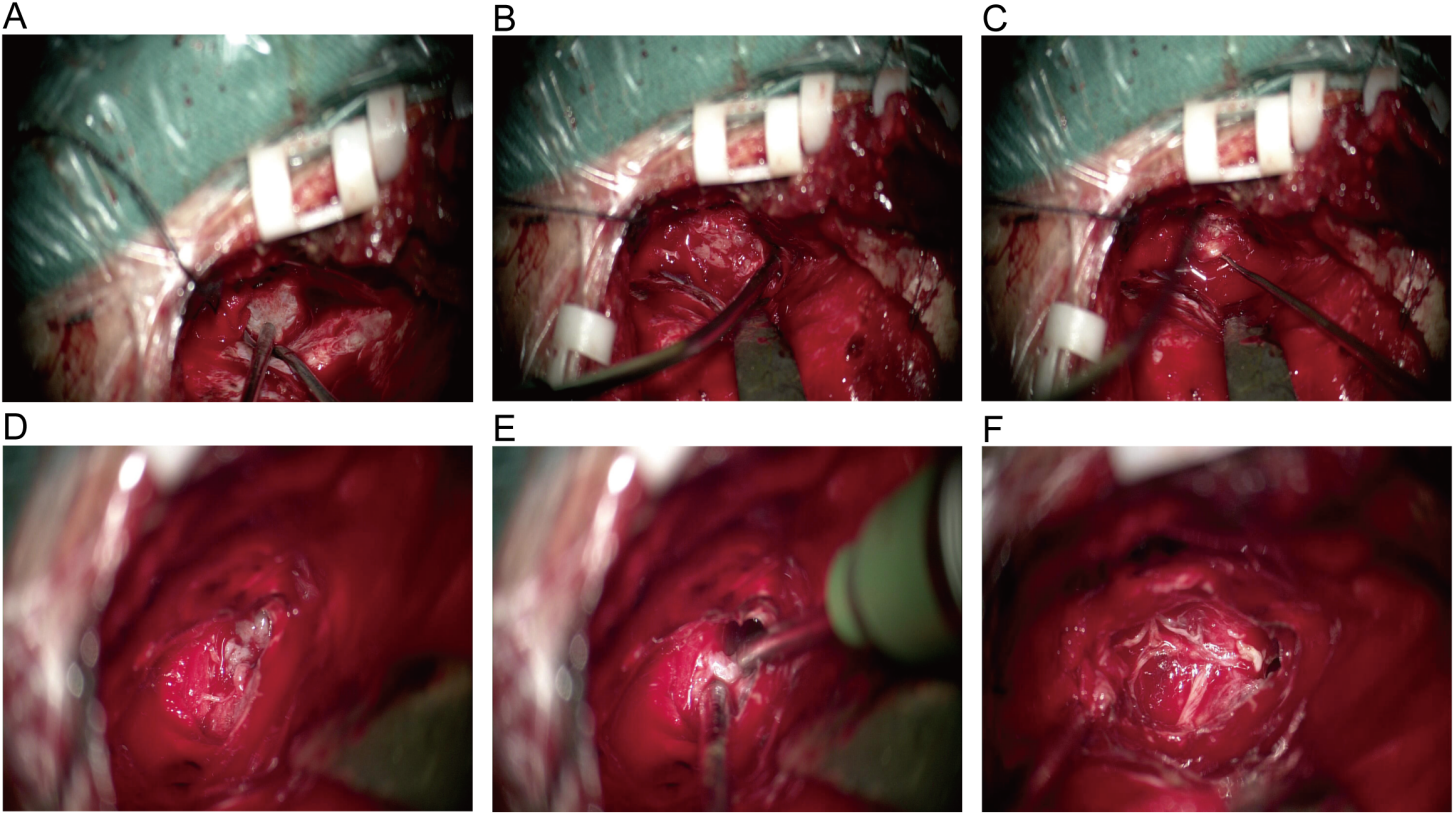
**(A)** Intraoperative exploration revealed intact tumor capsule and no adhesion to the dura mater. **(B)** Cut open the tumor capsule, the tumor texture is soft and gray white in color. **(C)** After removing a portion of the tumor tissue, expose the trigeminal nerve and strip the remaining tumor tissue along the nerve root. **(D)** The tumor grows through the trigeminal foramen and extends into Meckel’s cave. **(E)** Clear boundary between tumor and surrounding brain tissue, complete resection of tumors within Meckel’s cave. **(F)** After tumor resection, nerve protection was intact, and intraoperative exploration confirmed the origin of the EVN in the V cranial nerve.

A 53-year-old female patient presented to our neurosurgery department in June 2022 due to due to a 20-day history of right frontotemporal facial paresthesia and knifelike pain, accompanied by right facial twitching. The hearing test showed no abnormality. Magnetic resonance imaging (MRI) showed a 2.5 × 1.4-cm “dumbbell” enhancing lesion located in the cisternal segment of cranial nerve V and protruding into Meckel’s cave; the signal characteristics were suggestive of a trigeminal neurinoma (**Figure 1**). The Gd-DTPA-enhanced MRI revealed clear margins and substantial enhancement of the mass. The lesion was excised through the subtemporal middle cranial fossa approach, and the tumor can be completely exposed in the surgical field after grinding off the tip of the petrous bone. During the operation, the tumor was found to be cystic and solid, with soft texture and clear boundary with the Meckel’s cave, and it grew through the trigeminal foramen and extended into the Meckel’s cave. After opening the cerebellar tentorium, it could be seen that the tumor originated from the trigeminal nerve cistern segment. After cutting open the tumor capsule, it was found that the tumor tissue presented grayish white and lacked abundant blood supply. A portion of tumor was debulked using sharp dissection and bipolar cautery under the microscope, thereby exposing the trigeminal nerve and stripping the tumor along the nerve root, and completely removing the tumor tissue in Meckel’s cave (**Figure 2**).

**Figure 3:**
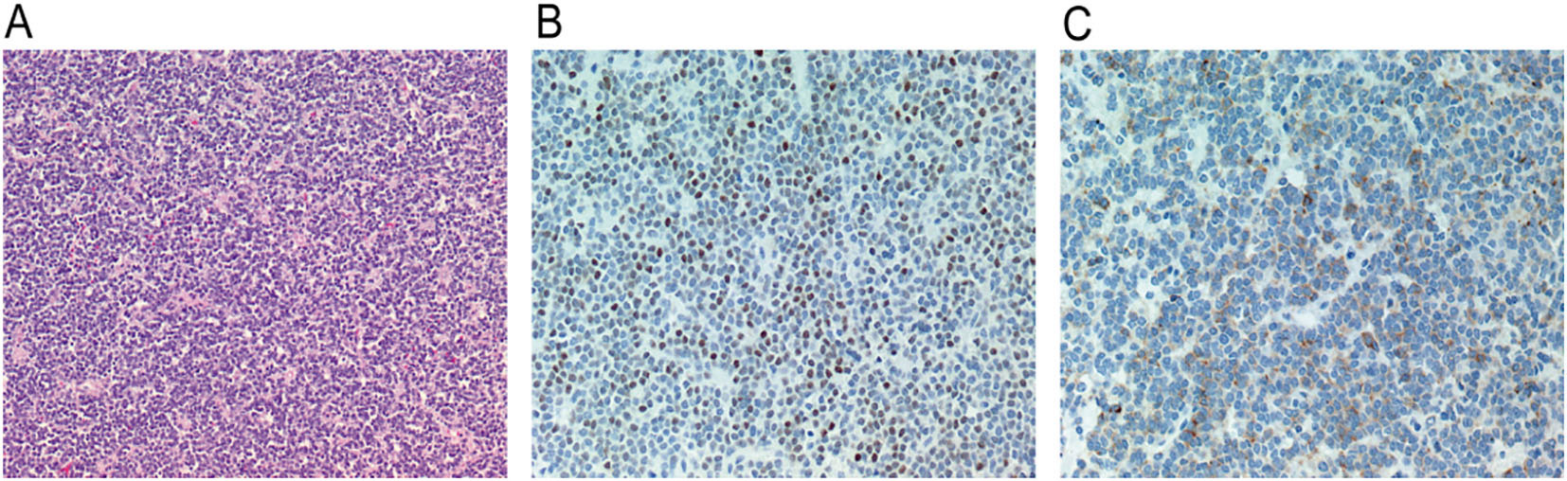
**(A)** Tumor cells were diffusely distributed in sheets, the size of the tumor cells was relatively uniform, the nuclei were oval to round, the cytoplasm was lightly stained, and mitotic figures were rare (hematoxylin and eosin, ×100). **(B)** Tumor cells express NEUN positive (original magnification, ×200). **(C)** Immunohistochemical stain for synaptophysin showing diffuse, strong reactivity in tumor cells (original magnification, ×200).

Pathological Findings. Histological examination revealed that the tumor cells were diffusely distributed in the form of sheets. The size of tumor cells was relatively uniform. The nuclei were oval to round and the cytoplasm was lightly stained, with rare mitotic Figures. The cells exhibited positive immunostaining for synaptophysin and NeuN (**Figure 3**). Additionally, the cells were strongly positive for CD56 and INI-1, and partially positive for CD99 and vimentin, while negative for EMA, S-100 protein, GFAP, SOX10, LCA, NSE, CgA, olig-2, and Brachyury. The Ki-67 index was high (> 5%).

**Figure 4:**
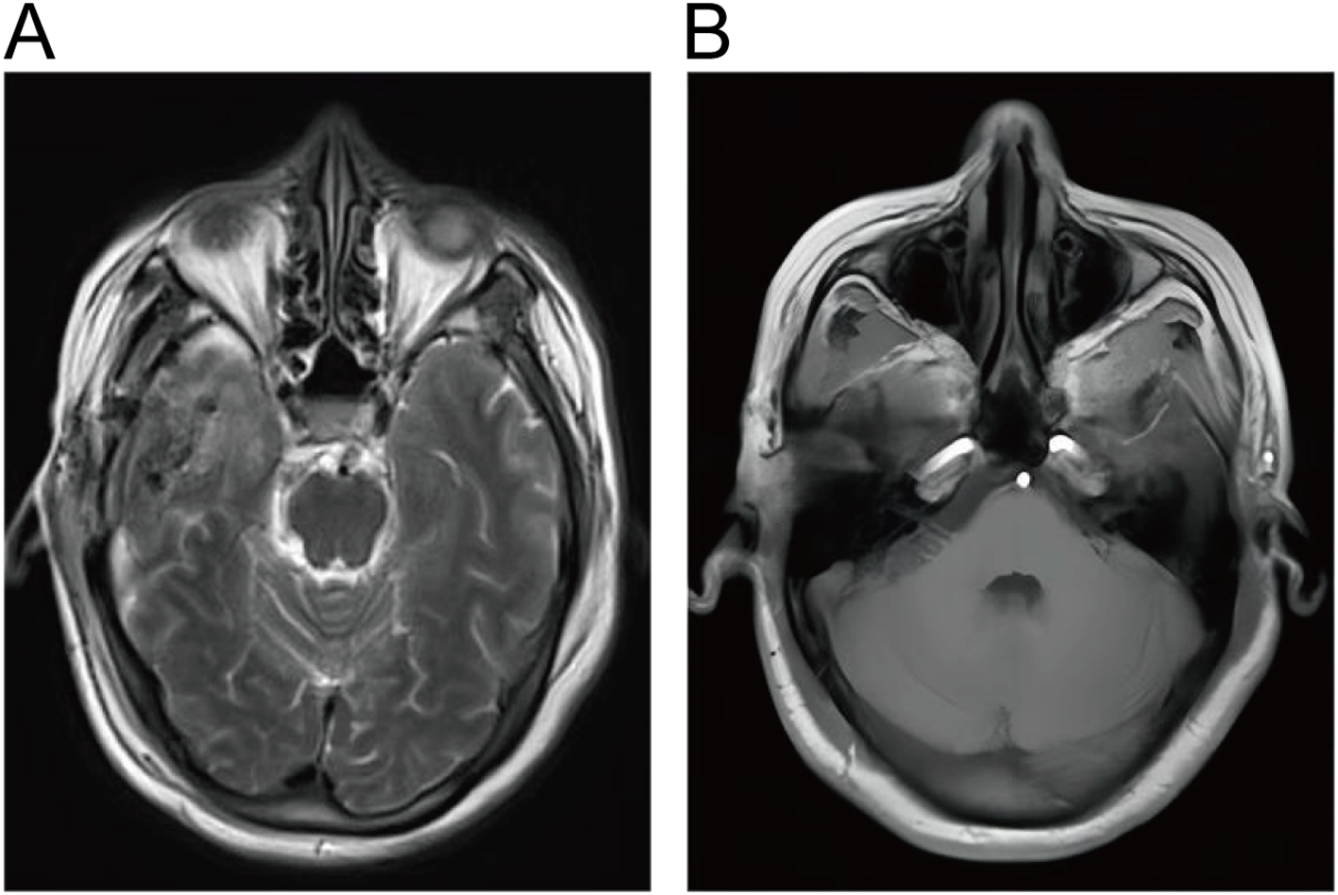
**(A, B)** Postoperative cranial MRI showed complete resection of EVN without residual lesions.

Treatment and Follow-up. Postoperative MRI showed that the tumor was resected. The patient recovered well postoperatively and did not require adjuvant therapy. One month postoperatively, facial knifelike pain and hemifacial spasm were improved significantly and there were no signs of tumor recurrence. One year after surgery, the patient underwent a follow-up examination and found improvement in facial sensation. MRI did not reveal any signs of tumor recurrence (**Figure 4**).

## 3 Discussion

CNs usually involve the lateral ventricles and septum pellucidum near the foramina of Monro. EVNs that occur in the brain parenchyma outside the ventricular system are rare (1), affecting almost 0.022 per 100,000 people (7). As reported, EVNs may appear in heterogeneous locations, including the cerebral lobes, sellar region, cerebellum, pons, brainstem, thalamus, hypothalamus, amygdala, basal ganglia, spinal cord, cauda equina, retina, pelvis, and ovaries (8). To date, merely two cases of EVNs arising from the cranial nerves have been reported, both originated from cranial nerve VIII (5, 6). In our patient, the tumor originated from the cisternal segment of cranial nerve V and extended through the trigeminal foramen into Meckel’s cave. To our knowledge, this is the first reported case of EVN originating from the trigeminal nerve.

Since EVNs occur in heterogeneous locations and their symptoms depend on the mass effect of lesions on surrounding tissues. Due to the absence of characteristic imaging features, EVNs are difficult to be differentiated from other intracranial tumors on imaging (4, 9). Our patient presented with right frontotemporal paresthesia and pain. Her MRI showed a dumbbell-enhancing lesion located in the cisternal segment of cranial nerve V and projecting into Meckel’s cave, similar to the findings of trigeminal neuroma. Histologically, CNs and EVNs typically show uniform, small, and round cells with clear cytoplasm in a background of nerve fibers (8), which is consistent with the microscopic observations in our case. Synaptophysin is the most reliable immunohistochemical marker of CNs and EVNs because of its strong immunoreactivity in nerve cells (10). Moreover, CNs and EVNs also frequently exhibit immunoreactivity to NeuN and neuron-specific enolase (11). In our case, the tumor was positive for synaptophysin and NeuN. Additionally, the tumor cells were strongly positive for CD56 and INI-1, and partially positive for CD99 and vimentin. At present, surgical resection remains the mainstay treatment of CNs and EVNs. Although gross total resection (GTR) is the preferred surgical option, subtotal resection and radiotherapy are sometimes necessary considering the proximity of the tumors to functionally important areas (12). In our case, the intraoperative findings revealed that the tumor was covered by capsules with sharp margins. The tumor was excised during the surgery. Although the Ki67 index was greater than 5%, we did not administer radiotherapy. The patient is on regular follow-up.

The origin of CNs and EVNs has not been clarified yet. Previously, CNs were considered to be purely neuronal tumors; however, emerging evidence suggests that CNs arise from undifferentiated precursor cells with neuronal and glial differentiation potential (13). Since EVNs are rarer and more diverse than CNs, the origin of EVNs from the peripheral nervous system (PNS) remains highly debated. Some experts have proposed that EVNs originate from bipotent progenitor cells in the periventricular interstitium, because of the potential of EVNs to differentiate into mature neurons or astrocytes (14). By contrast, some experts have also pointed out that EVNs are a component of the displaced CNS. Stephan et al. (15) reported a case of neurocytoma involving the cauda equina nerve roots, suggesting that the tumor may arise from the central stump of the nerve root or from the displaced CNS tissue in the peripheral segments of the nerve root. Finally, some studies have proposed that EVNs originate from the transitionnl zone between the CNS and PNS. Onguru et al. (5) reported a case of EVN that originated from the cochlear and vestibular portions of cranial nerve VIII, presumably from its CNS-PNS transitional zone. Similarly, we assumed that the tumor in our patient originated from the trigeminal root entry zone, the transitional zone of central and peripheral tissue compartments in the trigeminal nerve (16). In our patient, the tumor was located in the cisternal portion and extended into the Meckel cave near the trigeminal root entry zone. The initial segment of the nerve (root entry zone) contains glial cells, which are gradually replaced by peripheral Schwann cells in the transitional zone. The longest glial segments are located in the root entry zone of cranial nerve VIII (almost 10 mm), followed by the sensory part of cranial nerve V (almost 3 mm) and cranial nerve III (almost 1 mm) (17–19). Arnautovic et al. (20) found that the length of glial segments of these cranial nerves corresponded to the number of cases of extra-axial primary glial tumors (CN VIII, 8 cases; CN V, 3 cases; CN III, 1 case). Accordingly, it was speculated that oligodendrogliomas originated from the root entry zones of cranial nerves VIII and V. In line with this, Breshears et al. (21) first reported a case of glioblastoma involving the trigeminal nerve root entry zone in 2014 and revealed that glial tumors can occur in the root entry zones of cranial nerves VIII, V, and III. Moreover, two cases of neurocytoma arsing from cranial nerve VIII have also been reported. In our patient, the tumor originated from cranial nerve V. These findings support the hypothesis that neurocytomas of the cranial nerve originate from the CNS-PNS transitional zone. We speculate that neurocytomas may also arise from cranial nerve III, albeit very rarely.

## 4 Conclusion

In conclusion, EVNs originating from cranial nerves are extremely rare. At present, relatively little is known about the origin and pathobiological basis of the lesion location. Our case report of neurocytoma originating from cranial nerve V may provide new clinical information on the origin and pathological basis of lesion location in neurocytomas originating from cranial nerves.

## Data Availability

The original contributions presented in the study are included in the article/supplementary material. Further inquiries can be directed to the corresponding authors.
